# General Practitioners’ Perspectives on Digital Health Applications for Mental Disorders and Their Prescribing Behavior: Mixed Methods Study

**DOI:** 10.2196/78659

**Published:** 2026-01-06

**Authors:** Sandy Scheibe, Sandra Salm, Karola Mergenthal, Deborah Engesser, Esther Stalujanis, Susanne Singer, Pascal Kemmerer, Lena Dotzauer, Karen Voigt

**Affiliations:** 1 Department of General Practice, Faculty of Medicine and University Hospital Carl Gustav Carus, TUD Dresden University of Technology, Fetscherstraße 74, 01307 Dresden, Germany, 49 351-45889240, 49 351-4588836; 2 Institute of General Practice, Goethe University Frankfurt, Frankfurt am Main, Germany; 3 Medical Psychology—Neuropsychology and Gender Studies and Center for Neuropsychological Diagnostics and Intervention (CeNDI), Faculty of Medicine and University Hospital Cologne, University of Cologne, Köln, Germany; 4 Division of Epidemiology and Health Services Research, Institute of Medical Biostatistics, Epidemiology and Informatics (IMBEI), University Medical Center of the Johannes Gutenberg University Mainz, Mainz, Germany; 5 Department of Consultation-Liaison-Psychiatry and Psychosomatic Medicine, University Hospital of Zürich, Zürich, Switzerland; 6 Department of Psychology, Clinical Psychology and Psychotherapy–Methods and Approaches, Trier University, Trier, Germany; 7 Department of Quality of Life in Oncology, Comprehensive Cancer Center Mecklenburg-Vorpommern (CCC-MV), University Medical Centre Rostock, Rostock, Germany

**Keywords:** digital health, mobile apps, mental health, mixed methods, primary care, general practitioners

## Abstract

**Background:**

The high number of mental disorders poses challenges for health care systems. In 2020, digital health applications (DHAs) were introduced in Germany as a new form of health care financed by the statutory health insurance. They aim to detect, monitor, treat, or alleviate disease, injury, or disability. DHAs for mental disorders (DHA-MD) intend to improve outpatient care for patients with mental disorders. However, evidence on general practitioners’ (GPs’) perspectives on DHA-MD and their prescribing behavior is limited.

**Objective:**

This study aimed to analyze GPs’ perspectives on DHA-MD and their prescribing behavior in the care of patients with mental disorders.

**Methods:**

A mixed methods study was conducted (January-October 2024), including a Germany-wide online survey and qualitative interviews with GPs and medical assistants (MAs). Sampling was conducted in collaboration with German research practice networks, which distributed the study invitation to their affiliated GPs. The questionnaire as well as the interview guides for GPs and MAs was developed by the study team according to the Consolidated Framework for Implementation Research. Descriptive analyses of prescribing behavior and perceived need (measured on an 11-point scale) for DHA-MD were conducted, followed by multivariate regression analyses to identify predictors of prescribing behavior and perceived need for DHA-MD. The interviews with GPs and MAs were analyzed using qualitative content analysis according to Mayring.

**Results:**

A sample of 149 GPs participated, and 12 GPs as well as 5 MAs were interviewed. The median prescription frequency of DHA-MD per quarter was 1, whereas the median estimated need was 3. Working in a half digitized and half paper-based practice (odds ratio 5.133, 95% CI 1.695‐15.542) as well as working in a completely digitized practice (odds ratio 3.006, 95% CI 1.296‐6.969) positively predicted the prescribing behavior. The duration of GPs’ medical practice (*b*=−0.057; *P*=.01) negatively predicted the perceived need, while working in a group practice (*b*=0.980; *P*=.02) positively predicted the perceived need for DHA-MD. In the interviews, GPs and MAs reported that they valued DHA-MD as a temporary or supplementary option for bridging waiting times for psychotherapy and considered their effectiveness to be highly dependent on indication and patient adherence. Reported barriers of GPs according to DHA-MD included lacking knowledge about DHA-MD, missing effectiveness studies, and difficulties integrating them into existing care processes.

**Conclusions:**

GPs are reluctant to prescribe DHA-MD, as the need is considered to be low and their use is primarily seen as a temporary or supplementary treatment option rather than a stand-alone intervention. There are significant reasons for rejection and barriers that hinder prescription in primary care. Addressing these barriers and involving GPs as well as patients in future research are essential for the development of DHA-MD.

## Introduction

The global burden of mental disorders is high, affecting about 970.1 million people in 2019. In Germany, the lifetime prevalence of mental disorders in adults is 25.2% [1], which corresponds to a value in the midrange of international estimates. Mental disorders are among the disease groups with the highest burden for those affected [2,3], including reduced quality of life and functional capacity, inability to work, or early retirement, as well as an increased mortality [4-6]. In 2023, mental disorders accounted for 16.0% of incapacity to work days in Germany, and they were the leading cause of early retirement, accounting for 41.8% [7,8]. In addition, the indirect and direct costs arising from mental disorders are estimated to amount to more than €600 billion (approximately equal to US $696 billion) per year, exceeding about 4.0% of the gross domestic product across the European countries [9]. In Germany, the direct health care costs for mental disorders amounted to around €56.3 million (approximately equal to US $65.3 million) in 2020, showing a significant increase compared with 2015 (€42.7 million, approximately equal to US $49.5 million).

These findings highlight the need for adequate and accessible treatment. However, globally, the treatment coverage for mental disorders is low, suggesting a considerable degree of unmet treatment need [10,11]. Among treated cases with mental disorders, there is a long delay between onset and first treatment contact [10-13]—median waiting times for access to psychotherapy in European countries are more than 2 months [14]. In Germany, the recent psychotherapist act has potentially influenced the waiting times for the first appointment after initial consultation— being 2.6 before and 3.8 months after the reform [15].

To counteract the strained treatment situation, great hopes were being placed in digital health, such as mobile health (mHealth) apps. In 2020, Germany became the first country worldwide to grant statutorily insured individuals the right to use certain mHealth apps at the expense of health insurers. Afterward, other European countries such as France and Belgium have introduced approval procedures for digital health application (DHA)–equivalent health apps similar to the German model [16,17].

DHA are certified medical products, according to the European Medical Device Regulation, primarily based on digital functions to detect, monitor, treat, or alleviate disease, injury, or disability. For legal authorization as a medical device, DHA must demonstrate a positive health care effect (ie, improvement of disease symptoms, quality of life, health literacy, and feeling better involved in the treatment) to be permanently approved by the German Federal Institute for Drugs and Medical Devices (German, Bundesinstitut für Arzneimittel und Medizinprodukte [BfArM]) [18]. To date (October 2025), 56 DHAs are reimbursable by statutory health insurers. Mental disorders represent the largest group of indications for available DHA (29/56, 51.8%), including applications for treatment of insomnia, depression, anxiety disorders, or nicotine dependence (hereinafter referred to as DHA for mental disorders, DHA-MD) [19].

Health care professionals in the outpatient care sector play an important role in the implementation process, as reimbursement of DHA is possible when prescribed by physicians or psychotherapists [20-22]. Four years after the introduction of DHA, they were prescribed by 12.0% of physicians and psychotherapists [23]. The majority of DHA prescriptions were issued by general practitioners (GPs) [24].

Systematic reviews including studies from all over the world analyzing the implementation of digital health technologies in routine care identified several barriers faced by health care professionals, including technical limitations (eg, insufficient network coverage and lack of existing technologies or devices), insufficient expertise, and legal and ethical concerns (eg, privacy and security concerns and national legislation), as well as financial barriers (eg, high costs and inadequate remuneration structure). Reported facilitators included access to reliable information about digital health services, the perceived usefulness, and government monetary incentives [25-27]. However, studies that examine perspectives of health care professionals toward DHA, according to the definition of BfArM, are rare. Dalhausen and colleagues [20] conducted a mixed methods study to examine attitudes of GPs and psychotherapists toward DHA. The results indicated that GPs and psychotherapists expressed a generally positive attitude and openness toward DHAs. Attitudes and prescription intentions were significantly influenced by digital affinity, that is, GPs with a higher digital affinity were more likely to prescribe DHA. Age, practice type, and practice location were not associated with DHA prescriptions.

Another perspective is provided by Posselt and colleagues [21], who examined GPs’ key challenges in prescribing DHA-MD for patients with depression. They identified the following challenges: information gaps, insufficient knowledge about available information sources for DHA-MD, and difficulties in selecting patients suitable for DHA-MD use [21].

Previous studies on DHA-MD in Germany predominantly relied on qualitative data or focused on specific indications, such as depression. To date, no study combined quantitative and qualitative research methods to comprehensively analyze GPs’ perspectives on DHA-MD, across the whole spectrum of mental disorders. The aim of this study was therefore to analyze GPs’ perspectives on DHA-MD and their prescribing behavior in the care of patients with mental disorders.

## Methods

### Study Design

This study was conducted in Germany. We used a mixed methods convergent parallel design in which quantitative and qualitative data were collected and analyzed separately. The triangulation of the results took place during the interpretation phase by comparing and contrasting the findings of both strands to identify areas of convergence, divergence, or complementarity [28,29].

In the “Results” section, both data strands were first presented separately according to their respective methods, while in the “Discussion” section, the results were integrated and interpreted jointly along overarching themes [30], with qualitative results used to explain and deepen the understanding of quantitative results on GPs’ perspectives and prescribing behavior.

### Online Survey

#### Participants and Recruitment

Participants were practicing GPs (specialists in family or internal medicine) working in their own practices or as an employee in a practice, as well as clinical residents. The link to the online survey was distributed nationwide via email to German research practice networks and professional associations, as well as presented at public events for GPs in Germany. Subsequently, the research practice networks forwarded the invitation to their affiliated GPs, resembling a snowball sampling approach. Due to this strategy, it is not possible to determine the exact number of GPs who received the invitation to participate. The data were collected cross-sectionally through an anonymous online survey from January to October 2024.

#### Questionnaire

The questionnaire was developed by the study team according to the Consolidated Framework for Implementation Research model, which captures barriers and enablers in the implementation of interventions [31]. We conducted a pretest of the questionnaire with 6 GPs who verified the relevance and completeness of the items. Based on the feedback of GPs, the wording of 1 question was revised to improve clarity. The overall structure and methodology of the questionnaire were retained after pretest.

#### Demographic and Practice Characteristics

We collected sociodemographic information (ie, gender, age, and duration of practicing as GP) and characteristics of GP practices (ie, type of practice [single vs group practice], location [urban-rural], number of treated patients per quarter, and extent of digitization in practice). To determine the extent of digitization in practice, GPs stated on a 5-point scale whether their communication with colleagues in outpatient care and patients is “almost completely digitized” (0) or “almost completely paper-based” (4). For regression analyses, we recoded this variable into 3 categories: “digitized” (0‐1), “half digitized and half paper-based” (2), and “paper-based” (3-4).

#### Prescription Behavior and Perceived Need for DHA-MD

Participants were asked how many patients they had prescribed a DHA (all indications) or a DHA-MD in the last quarter. Since a large proportion of participants reported having prescribed no DHA-MD at all, we recoded this variable into a binary measure (prescription=yes or no) to facilitate analysis using logistic regression. A prescription was considered present if at least 1 patient had received a DHA-MD during the last 3 months. The perceived need for DHA-MD for patients was estimated on an 11-point scale, ranging from “very low” (0) to “very high” (10).

#### Statistical Analysis of the Survey Data

Quantitative data were analyzed using SPSS (version 30; IBM Corp). Descriptive statistics were presented as mean values with standard deviations for metric-scaled variables and as percentages and frequencies for nonmetric-scaled variables in order to describe the study sample.

The following variables were the predictors of the regression analyses: gender (male or female), age (years), duration of GP practice (years), type of practice (single practice or joint practice), practice location (city or rural area), and degree of digitization in practice (paper-based, or half digitized and half paper-based, or digitized). Correlations between predictor variables were examined to rule out multicollinearity. In cases of high correlation (*r*>0.80) [32], a decision was made to exclude one of the variables from the analysis based on theoretical considerations and relevance to the research question. Dependent variables were analyzed via multivariate analyses using logistic regression modeling for prescribing behavior and linear regression modeling for perceived need of DHA-MD. Missing values were not imputed. Participants with missing data were excluded from the respective analyses. The Gauss-Markov assumptions were tested as prerequisites for the multiple linear regression of perceived need of DHA-MD. Model fit was assessed using Nagelkerke’s *R*² for the logistic regression model and adjusted *R*² for the multiple linear regression model. The significance level was set at α≤.05.

### Qualitative Study

#### Participants and Procedures

Invitations for the telephone interviews were distributed via the German research practice network “SaxoForN” [33], which subsequently forwarded the invitation to their affiliated GPs (snowball sampling). Medical assistants (MAs) were also invited to participate in qualitative interviews, as they are closely involved in administrative processes of GP practices and can provide organizational and time relief for GPs in the German health care system [34]. Previous research has shown that patients have a high level of trust in MAs [35], who often serve as the first point of contact for questions or issues that may not be raised during the GP consultation—possibly also with regard to the prescription and use of DHA. Moreover, as certain tasks in primary care are delegated from GPs to MAs [35], we aimed to explore which concrete tasks they take over in relation to DHA—such as assisting in administrative procedures, supporting patient onboarding, or addressing patient inquiries during the usage phase. Including MAs in the qualitative study enabled us to explore their role in the implementation process and capture patient-related challenges they identified, which may extend beyond the GPs” perspective. Only MAs who worked in GP practices with DHA prescriptions were interviewed to ensure whether they were already familiar with the concept of DHA. Interviews were conducted with all interested GPs and MAs who had registered to participate. Telephone interviews took place between July and October 2024 and were conducted by 2 researchers (SaS and SSch) of the study team. Both interviewers were female and health scientists. They had no prior relationship with the participants.

#### Telephone Interviews

Semistructured interview guides for GPs and MAs were developed according to the Consolidated Framework for Implementation Research framework [31]. The interview guides were pretested by 2 GPs in December 2023. Feedback revealed that no changes were deemed necessary in terms of methodology, structure, or questions.

The interviews with GPs started with an opening question, which transitioned to the following 3 key topics: experiences with DHA (overall; for DHA-MD), attitudes toward DHA-MD, and implementation factors and conditions for the use of DHA-MD in outpatient care. MAs were asked about their tasks related to DHA in GP practice, frequently asked questions of patients related to DHA, and feedback of patients to DHA use.

If necessary, the interviewers asked further questions to go more in-depth on the information the participants provided. Despite the interview guide, participants were encouraged to talk freely without too much interruption from the interviewers. Theoretical saturation was assessed iteratively during data collection by the interviewers and was deemed achieved when interviews no longer yielded novel insights and a sufficient heterogeneity of perspectives had been captured. To ensure a transparent and consensual process, regular team meetings were held to discuss emerging themes and determine the point of saturation. The telephone interviews were audio-recorded and transcribed verbatim. To ensure data protection, all personal data were pseudonymized in the transcription process.

#### Qualitative Content Analysis of the Interviews

The interviews with GPs and MAs were analyzed using qualitative content analysis according to Mayring [36]. The coding schemes for the interviews were developed using a deductive-inductive approach, based on the previously developed interview guides. Two researchers (SSch and SaS) analyzed the interviews independently with use of the software MAXQDA (version 2020; VERBI Software). The results were subsequently cross-compared, whereby disagreement was discussed until consensus was reached. If necessary, a third senior researcher was consulted.

### Ethical Considerations

This study was approved by the ethics committee of State Medical Association of Rhineland-Palatinate (no. 2023‐17268), ethics committee of Dresden University of Technology (no. SR-EK-418092023), and the Medical Faculty of Goethe University Frankfurt am Main (no. 2023‐1505). Participation in the online survey was anonymous and therefore required no consent for the use of data according to German law. Before conducting the telephone interviews, written informed consent was obtained from all participants. All interview data were collected in pseudonymized form. Any identifying information mentioned during the interviews (eg, names of persons, places, or organizations) was anonymized during transcription and replaced with nonidentifiable character strings to prevent any possibility of reidentification. Participants received a compensation of €50 (approximately equal to US $58) for participating in the interview.

## Results

### Online Survey

#### Characteristics of the Study Population

A total of 149 participants completed the questionnaire, of whom 47.7% (71/149) were male and 52.3% (78/149) were female. As shown in Table 1, the mean age of the respondents was 50.7 (SD 10.5) years. The mean work experience of GP was 15.2 (SD 9.9) years. Most respondents were practice owners (111/149, 74.5%) and located in urban areas (122/148, 82.4%) with various community sizes (Table 1). About half of the respondents were active in single practices without physician colleagues (74/145, 51.1%), while the other half (71/145, 48.9%) worked jointly with at least 1 colleague.

**Table 1.** Description of study population.

Variables	Participants
Categorical variables, n (%)	
Sex, n=148	
Male	71 (47.7)
Female	78 (52.3)
Position in practice, n=149	
Practice owner	111 (74.5)
Employed	31 (20.8)
Clinical residents	7 (4.7)
Type of practice, n=145	
Single practice	74 (51.1)
Joint practice	71 (48.9)
Practice location, n=148	
Large city (>100,000 inhabitants)	50 (33.8)
Medium-sized city (20,000‐100,000 inhabitants)	35 (23.6)
Small city (5000‐20,000 inhabitants)	37 (25.0)
Rural community	26 (17.6)
Communication with patients and colleagues in practice, n=141	
Digitized	26 (18.4)
Half digitized and half paper-based	62 (44.0)
Paper-based	53 (37.6)
Treated patients per quarter, n=145	
<1000	18 (12.2)
1000‐1999	75 (50.6)
2000‐2999	33 (22.3)
>3000	19 (12.9)
Numerical variables (mean SD)	
Age (years), n=149	50.7 (10.5)
Duration of GP^a^ practice (years), n=148	15.2 (10.0)

a GP: general practitioner.

#### Prescribing Behavior and Perceived Need for DHA-MD

Of the participating GPs, 65.7% (90/137) prescribed at least 1 DHA in the last quarter. The median prescription frequency for DHA (for all indications) per quarter was 2 (IQR 0‐5) and for DHA-MD, it was 1 (IQR 0‐2). Nearly half of the respondents (68/137, 49.6%) did not prescribe any DHA-MD in the last quarter. The median estimated need was 3 (IQR 1‐5).

The multivariate logistic regression included the predictors gender, age, duration of GP practice, type of practice, practice location, and degree of digitization in practice. Correlations between predictor variables indicated a high correlation between age and duration of GP practice (*r*=0.87; *P*<.001), justifying the decision to exclude age as a predictor in the regression model. All other correlations were low (*r*<.25). Working in a half digitized and half paper-based practice (odds ratio 5.133, 95% CI 1.695‐15.542) as well as working in an almost completely digitized practice (odds ratio 3.006, 95% CI 1.296‐6.969) positively predicted prescribing behavior (Table 2). Hosmer-Lemeshow test indicated a good model fit (*χ*²_8_=12.43; *P*>.05) for the logistic regression model. The model explained 13.9% of the variance (Nagelkerke’s *R*²=0.139).

The multivariate linear regression analysis included the same predictors as in the logistic regression analysis. The Breusch-Pagan test indicated no heteroscedasticity (*P*=.45). The duration of GPs’ medical practice (*b*=−0.057; *P*=.01) negatively predicted the perceived need, while working in a group practice (*b*=0.980; *P*=.02) positively predicted the perceived need for DHA-MD (Table 3). The model explained 8.2% of the variance (corrected *R*^2^=0.082; *P*=.009).

**Table 2.** Multivariate logistic regression analysis for general practitioners’ prescribing behavior (yes/no) of digital health applications for people with mental disorders (n=133).

Predictor	OR^a^ (95% CI)	*P* value
Sex		
Female	Reference category	
Male	0.608 (0.285‐1.296)	.20
Duration of GP^b^ practice	0.992 (0.953‐1.032)	.69
Type of practice		
Single practice	Reference category	
Joint practice	1.788 (0.835‐3.830)	.14
Practice location		
City	Reference category	
Rural area	1.177 (0.556‐2.490)	.67
Degree of digitization in practice		
Paper-based	Reference category	
Half paper-based/half digitized	**5.133**^**c**^ **(1.695‐15.542)**	**.004**
Digitized	**3.006 (1.296‐6.969)**	**.01**

a OR: odds ratio.

b GP: general practitioner.

c Values in boldface indicate statistical significance.

**Table 3.** Multivariate linear regression analysis for general practitioners’ perceived need of digital health applications for people with mental disorders (n=135).

Predictor	*b* value	95% CI	*P* value
Sex			
Male	Reference category		
Female	0.350	−0.498 to 1.199	.45
Duration of GP^a^ practice	**−0.057** ^**b**^	**−0.102 to −0.012**	**.01**
Type of practice			
Single practice	Reference category		
** **Joint practice	**0.980**	**0.138 to 1.822**	**.02**
Practice location			
City	Reference category		
Rural area	0.227	−0.629 to 1.082	.60
Degree of digitization in practice			
Paper-based	−0.823	−1.775 to 0.128	.09
Half paper-based/half digitized	Reference category		
Digitized	0.257	−0.888 to 1.402	.66

a GP: general practitioner.

b Values in boldface indicate statistical significance.

### Telephone Interviews

Interviews were conducted with 12 GPs and 5 MAs. The interviews with GPs varied in duration between 18 and 38 minutes (mean duration 25 minutes), and the interviews with MAs varied between 7 and 13 minutes (mean duration 9 minutes).

#### GPs’ Experiences With DHA-MD

All interviewed GPs stated that they had *already prescribed DHA* for different indications in practice, including DHA-MD addressing mental disorders. *Reasons for prescribing *DHA-MD to patients were positive experiences with individual DHA-MD, patient request, the ability for GPs to view the contents before prescribing (GP test access), and the opportunity to offer patients a treatment alternative to existing therapy options.

GPs’ e*xperiences with health insurance companies*, as well as with the *activation process of DHA-MD*, were mixed. Some GPs mentioned occasional problems with health insurance companies, including long waiting times for prescription processing, prescriptions being completely rejected by individual health insurance companies, and technical problems when redeeming them. Others stated that the processes with health insurance companies and the activation process of DHA-MD were straightforward, and patients usually gained access to DHA-MD within a few days (Multimedia Appendix 1).

Feedback from GPs regarding their *experiences with the utilization of DHA-MD* by patients was heterogeneous. Some GPs reported positive user experiences of patients. Others, however, felt that only a certain group of patients benefited from DHA and used it as intended —namely, those who were particularly motivated and willing to actively engage in the management of their mental health condition. In a few cases, GPs reported that they issued prescriptions, but these were not redeemed at all by patients. GPs stated that patients carefully consider whether treatment with DHA-MD fits their personal context and preferences and whether the content of the DHA-MD aligns with their needs. In addition, GPs reported a lack of integration in the treatment with the DHA-MD, making it difficult for them to monitor patient adherence and the treatment progress.

#### GPs’ Attitudes to DHA-MD

Regarding the *assessment of the effectiveness of DHA-MD*, 4 different groups emerged in the analysis. The first group of GPs, who considered *DHA-MD to be effective and without side effects*, emphasized that DHA-MD would have stabilizing effects on patients by providing validated knowledge about their mental disorder and is especially helpful in bridging the waiting time for psychotherapy appointments. GPs further stated that DHA-MD could reduce physician-patient contacts and, in some mild cases, make psychotherapeutic treatment no longer necessary. The second group of GPs stated that DHA-MD *effectiveness depends on the patient characteristics*. Patients must be motivated and should use the DHA-MD as prescribed by the manufacturers to achieve sufficient effectiveness. The third group of GPs declared that DHA-MD *effectiveness depends on the indication*. According to the GPs, DHA-MD would be effective for conditions where cognitive-behavioral therapy is effective, such as anxiety disorders, but less so for borderline personality disorders. In addition, GPs mentioned that providing proof of effectiveness might be more difficult for certain mental health conditions because it cannot be measured as objectively as in other conditions (eg, weight loss in obesity). The fourth group of GPs expressed that evidence-based statements about the effectiveness of DHA-MD cannot be made due to *insufficient evidence*. They highlighted the lack of long-term studies and large-scale cohort studies, including subgroup analyses, to assess effectiveness in different patient groups.

With regard to *the importance of DHA-MD* in primary care, GPs mentioned societal benefits, meaning that the treatment with DHA-MD could reduce the use of antidepressants, minimize sick leave, and ease the burden on GP practices by reducing patient-physician contacts.

Regarding the question of how GPs assessed the potential of DHA-MD in the *collaboration between GPs and psychotherapists*, 2 different groups emerged. The first group of GPs emphasized that there has been little exchange between GPs and psychotherapists so far, and they believed that this situation would not change even with the development and integration of these innovative digital technologies into patient care. A second group of GPs could imagine that both groups of health care providers would be included in the DHA-MD, allowing them to simultaneously track the treatment process and promote interdisciplinary collaboration.

GPs’ assessment of *the perceived need for DHA-MD in primary care* was heterogeneous. On the one hand, GPs stated that there is a need for DHA-MD, justified particularly due to an increase in mental disorders, especially among younger adults, and as a consequence of the COVID-19 pandemic. In addition, they reported that DHA-MD would be helpful if they addressed a problem in general practice—for example, by providing treatment for patients in need of psychotherapy who face long waiting times, thereby alleviating pressure on GP practices. However, GPs also highlighted that developing new DHA-MD for indications already covered by existing products is unnecessary, as the market is becoming increasingly opaque, and they lack the time in their daily practice to inform themselves comprehensively about new products. Instead, they advocated in the interviews for a reevaluation and regular improvement of the quality of existing DHA-MD. On the other hand, GPs stated that the evidence for practical use of DHA-MD is still insufficient and that these digital applications would not be necessary if there were enough psychotherapy places and enough medical capacity that the conversations with patients could be partly conducted by GPs themselves.

#### MAs’ Experiences With DHA

Overall, MAs’ *experiences with DHA* were heterogeneous (Multimedia Appendix 2). All interviewed MAs stated that DHA had *already been prescribed* in the GP practices where they worked, including DHA for musculoskeletal disorders, mental disorders, and metabolic disorders. According to MAs, their main *sources of information about DHA* were the internet, manufacturer advertising, specialist journals, and test access. However, all MAs reported that they *lacked sufficient knowledge* about the contents and functionalities of DHA and expressed a need for increased public relations work and training in order to better assess the functionality of DHA, as well as the suitability for individual patients. MAs also reported *challenges in selecting patients suitable for DHA-MD use* because patients often do not talk openly about their mental disorder due to feelings of shame.

MAs evaluated *the importance of DHA* in primary care differently. On the one hand, DHAs were perceived as an innovative care solution, particularly for bridging waiting times for psychotherapy and as a supportive therapy. On the other hand, doubts were expressed about their usefulness, since personal contact to a GP or a psychotherapist was considered essential for mental disorders. With regard to the *integration of DHA into GP practice*, MAs reported that implementation in rural areas with a predominantly older patient population is difficult because they tend to be less tech-savvy. Most MAs reported not taking on any *tasks related to DHA* in GP practice. In some cases, there were organizational tasks for MAs in relation to DHA, for instance, preparing flyers for interested patients or reminding GPs to use the billing code in the reimbursement process when a DHA was prescribed. Most MAs reported that they had not been asked any *questions by patients in connection with DHA*. There were single questions regarding the functionality of DHA, the necessity to use, and if patients specifically requested a DHA.

According to MAs, detailed questions of patients were addressed directly with the GPs during the consultations. Regarding the *feedback of patients to DHA*, which was shared with MAs, three different groups emerged: (1) positive feedback, (2) negative feedback, and (3) no feedback received. The first group reported positive user experiences. According to MAs, younger patients who were highly motivated to use DHA benefit particularly and receive support in dealing with their own disorder, as well as making sustainable lifestyle changes. The second group reported negative feedback in detail that patients did not get along with the use of DHA. There was a third group of MAs who stated that they did not receive any feedback from patients because this was discussed exclusively during consultations between the GP and the patient.

## Discussion

### Principal Findings

Our study aimed to analyze GPs’ perspectives on DHA-MD and their prescribing behavior in the care of patients with mental disorders. The study is the first in Germany to show that while the majority of participating GPs have already prescribed DHA, they tend to prescribe DHA-MD only selectively in primary care and perceive their need as low. The importance of DHA-MD is particularly seen in bridging waiting times for psychotherapy. According to GPs, there are considerable reasons for rejection as well as barriers that hinder prescription.

### Prescribing Behavior

The proportion of GPs who have already prescribed DHA in our study was 65.7%, which was even higher than that in other studies from various countries (7.9% [20], 31.0% [37], and 50.0% [38]). Current billing data from German health insurance companies indicated a steady increase in DHA prescriptions with subsequent patient usage since 2020, rising from 41,000 to 209,000 in 2023 [24]. The relatively low prescription rate of 7.9% in the study by Dalhausen et al [20], conducted in Germany, may be explained by the study period, as the concept of DHA was still relatively new at this time. In contrast, the 50.0% rate reported in an Australian study [38] reflects a different health care context and a broader focus on mHealth app use rather than DHA-specific prescriptions. In addition, in Australia, there is no national reimbursement system for mHealth apps, unlike in Germany, meaning that patients have to privately fund these health applications [38].

Focusing especially on DHA-MD, half of the participating GPs in our study had prescribed at least 1 DHA-MD in the last 3 months. Mental disorders are one of the most common reasons for consultation in general practice [39]. In this context, billing data from German health insurance companies revealed that the majority of DHA-MD prescriptions (45%) were issued by GPs [24]. The interviews with GPs provided a possible explanation, indicating that GPs prescribe DHA-MD particularly to offer their patients an alternative treatment option and to bridge waiting times for psychotherapy.

Furthermore, existing literature highlighted that health care professionals are more likely to adopt a new technology if they perceive it as beneficial for their own work or their patients [22,40]. Consistent with these findings, GPs in our study have not only emphasized patient-related advantages of DHA-MD, such as improved patient education and the low-threshold access, but also pointed to disadvantages including delayed physician-patient interaction and adherence problems, which could explain the nonprescription rate as well as the low median prescription frequency of DHA-MD in our study. A potential selection bias toward increased participation of DHA critical GPs is also conceivable.

The multivariate logistic regression analysis identified the degree of digitization as a significant predictor of prescribing behavior, which aligns with the findings by Dalhausen et al [20] and international systematic reviews [22,40,41] on the adoption of digital health technologies, showing that health care professionals with greater digital affinity and experience held significantly more positive attitudes and were more likely to adopt digital technologies in practice. A possible explanation could be that GPs working in practices with a higher extent of digitization may experience fewer barriers to integration and greater confidence in practical benefits of DHA-MD.

However, both of our regression models showed limited explanatory power, suggesting that additional variables not included in the analyses may also contribute to explain GPs’ prescribing behavior and the perceived need. Future studies may therefore include other established predictors of digital health utilization, such as previous training, digital skills, general acceptance of and attitudes toward technology, or knowledge and beliefs about the intervention [42-45], to further elucidate these underlying mechanisms.

### GPs’ Perspectives on DHA-MD

About two-thirds of the survey respondents assessed the need for DHA-MD as low, which corresponds to a German study by Wangler and Jansky [46] on attitudes and experiences of GPs, indicating that some GPs refrain from prescribing DHA, considering their contribution to the improvement of health minimal. Our interviews revealed reasons for rejection of DHA-MD, including lacking knowledge about available DHA-MD, missing evidence on DHA-MD effectiveness, and limited integrability into existing care processes. These barriers align with a German systematic review on incentives for DHAs among physicians and psychotherapists [27], and an international systematic review on digital health services for musculoskeletal conditions in primary care [26], which additionally identified barriers related to data security and protection, the organizational workload, and the negative impact on the doctor-patient relationship. As in our interviews, high costs, inadequate reimbursement, missing financial incentives, and unclear liability risks were further reported as barriers [27]. A possible solution to these perceived barriers could be the implementation of “digital navigators” [47]—specially trained MAs who support health care professionals by evaluating available DHA, selecting suitable applications for patients, and preparing app-generated data for clinical decision-making. However, evidence on acceptance by health care professionals and feasibility of implementing these digital navigators in outpatient care has not yet been published [47], and further randomized controlled studies are needed to evaluate (cost)-effectiveness.

Our qualitative interviews yielded mixed perceptions of the need for DHA-MD. On the one hand, GPs stated that there would be no need for DHA-MD if sufficient psychotherapy places or adequate medical capacity were available. On the other hand, the majority of interviewed GPs reported a need for DHA-MD, particularly in light of the perceived increase in the number of patients with mental disorders due to the COVID-19 pandemic. There are no published comparable studies available, which quantify the perceived need of DHA-MD from the perspective of GPs, so our results close this evidence gap.

Our study found that perceived need declined with increasing years of GP experience. Due to the high correlation between age and duration of GP practice, age was not included as a predictor in the linear regression analysis. However, as these 2 variables are strongly related both statistically and conceptually, and evidence on the influence of duration of GP practice on the perceived need is lacking, available studies examining age-related effects on attitudes and health care technology adoption may offer valuable context for interpreting our findings. In accordance with our results, available literature showed that, in Germany, younger GPs rate DHA more positively than older GPs [20,48]. This age-related trend is also reflected in international studies from Australia [49] and Brazil [50] and could be explained by the fact that younger physicians are less hesitant to integrate new technologies into their practice [51].

The linear regression analysis revealed that GPs working in group practices rated the need for DHA-MD higher than those in single practices, which is in line with a systematic review from the United States on the adoption and use of health information technology in physician practice organizations. The results showed that compared with single practices, groups with 4-6 physicians were more likely to have an electronic medical record [52,53]. Peer influence and the organizational culture may contribute to this finding. Pollack and colleagues [54] showed in their study that social contagion among physicians has a significant influence on technology adoption—the more closely and frequently physicians interact with colleagues who also use a specific technology, the more likely it is that they will use it themselves. Furthermore, the international systematic review by Police et al [52] highlighted that a lack of commitment to technology integration, along with an organizational culture resistant to change, significantly impedes technology integration and utilization.

While our results indicated that GPs and their MAs preselect patients for the use of DHA-MD, the actual utilization is shaped by patients’ social determinants, which may limit access for certain patient groups and lead to systematic differences in digital participation (def. “digital divide”) [55,56]. According to the World Health Organization’s scoping review, people with greater health care needs, older adults, and marginalized groups are less likely to benefit from digital health interventions. In contrast, younger individuals with higher socioeconomic status living in urban areas tend to experience more positive effects when using these digital technologies [57]. These findings underline the necessity of future effectiveness studies on DHA-MD that incorporate subgroup analyses to assess not only which patient groups benefit most but also which may experience adverse effects or even harms.

To mitigate these disparities and ensure equitable access, it is crucial to integrate both health care providers and patients in the development of DHA-MD through a participatory approach, for instance, Co-Creation, to address their perceived barriers and to ensure that these technologies are designed, developed, and implemented within the specific contexts in which they will be applied [58]. In psychiatric care, this participatory process is particularly valuable to address the complex needs and challenges faced by users in their everyday lives. A participatory development process can significantly increase the acceptance and trust of both users and health care providers, who prescribe DHA-MD to their patients.

### Strengths and Limitations

Our study provides information to understand perspectives of GPs on DHA-MD and their prescribing behavior more comprehensively. By using a mixed methods design, we were able to triangulate quantitative data identifying broader trends with qualitative data that provide a more detailed and heuristic understanding of individual GP and MA perspectives. This methodological approach ensured that our findings are relevant for clinical practice, as well as for other health care stakeholders (eg, DHA manufacturer, BfArM). Although conducting an anonymous online survey may have introduced self-selection bias—with participation potentially skewed toward individuals with preexisting interest or strong opinions about DHA-MD—and qualitative interviews primarily attracted already interested participants, the mixed methods design addressed these limitations. The combination of quantitative and qualitative data allowed validation and mutual supplementation of the data. This integration mitigated the biases inherent in the individual methods and strengthened the robustness and practical relevance of the overall findings. However, a limitation of the qualitative study is that we conducted only 5 interviews with MAs. These interviews were intended to understand MAs’ role in the prescription process and use of DHA and to capture perspectives that might extend beyond those of GPs, for example, questions or issues raised by patients to MAs that are not discussed during GP consultations. However, most of the interviewed MAs reported no involvement in DHA prescription or follow-up. Therefore, these findings should be interpreted as exploratory. With regard to generalizability, it is important to acknowledge that our findings are embedded within the specific context of DHA in Germany. The German DHA framework provides a distinct legal foundation for the prescription and reimbursement of such applications. Nonetheless, the results may also hold relevance for other countries that have recently implemented comparable initiatives, including France, Belgium, and the United Kingdom. However, differences between national health care systems and reimbursement structures may constrain the direct transferability of our findings. Consequently, further international research is warranted to explore whether the patterns identified in our study can be replicated in other health care contexts. As the study used a cross-sectional design, it is inherently limited in its ability to infer causal relationships between the variables analyzed, such as between prescription frequency, digital affinity, and age.

### Conclusions

DHA-MD are currently prescribed cautiously by GPs, and their perceived need for patients with mental disorders is considered low, as reflected in the relatively high nonprescription rate observed in our study. GPs primarily justify the prescription of DHA-MD as a temporary solution to bridge waiting times for psychotherapy appointments or as a supplementary therapy option, rather than as a stand-alone intervention.

According to GPs, there are reasons for rejection as well as considerable barriers, primarily related to the structural framework of the DHA concept, which hinder prescription of DHA-MD in primary care. Given GPs’ key role in the prescription process, addressing both their perceived barriers and those of patients, as end users, is essential for the development of DHA-MD. One possible solution could be to actively involve both patients and health care providers in the development of DHA-MD through a Co-Creation approach to ensure that DHAs are need-related and designed within the specific health care settings in which they are used.

As the digital health care landscape continues to evolve rapidly—driven by technological advancements and shifting health care needs that frequently reshape regulatory frameworks and the availability of DHA-MD—ongoing research on GPs’ perspectives on DHA-MD is essential. In particular, future effectiveness studies are needed to objectively evaluate not only which patient groups benefit and which may even be harmed when using DHA-MD but also where alternative therapy approaches (eg, primary or psychotherapy care) are more effective.

## Supplementary material

10.2196/78659 Multimedia Appendix 1 Results of interviews with general practitioners.

10.2196/78659 Multimedia Appendix 2 Results of interviews with medical assistants.

## Abbreviations

AI: artificial intelligence
BfArM: German Federal Institute for Drugs and Medical Devices (German: Bundesinstitut für Arzneimittel und Medizinprodukte)
DHA: digital health application
DHA-MD: digital health application for mental disorder
GP: general practitioner
MA: medical assistant
mHealth: mobile health
