# Supplementary material for: General Practitioners’ Perspectives on Digital Health Applications for Mental Disorders and Their Prescribing Behavior: Mixed Methods Study
Source: JMIR Ment Health. 2026 Jan 6;13:e78659. doi: 10.2196/78659 (PMC12774394; doi:10.2196/78659)
Supplement: Multimedia Appendix 1 [file mental-v13-e78659-s001.docx]

Multimedia Appendix 1. Results of interviews with general practitioners.

| **Main category** | **Subcategory** | **Example quote** |
| --- | --- | --- |
| **Experiences with DHA-MD** | - **Already prescribed in practice** - **Experiences with health insurance companies** - **Experiences with the activation process** - **Experiences with the utilization** - **Experiences with the prescription** | *“Well, I have prescribed DHA for all kinds of psychiatric and psychosomatic disorders, of course, especially for depression, anxiety disorders and panic disorders.” (DD_GP08*)*  *“Actually, it works quite well. If the patients are open-minded, it usually works if you tell them that they don't have to hand it in personally at the health insurance company or send it by post. Most of them, well, health insurance companies now have an app where they can simply take a photo of it, and then they usually had the activation code just one or two days later. That it actually went quite smoothly” (DD_GP08)*  *“And one of them also had some kind of technical difficulties with the activation code and the health insurance and stuff like that. But I can't remember the details anymore.” (FFM04*)*  *“Well, yes. The feedback from patients is very different. There are patients who cope with [the DHA-MD] very well. They praise it very much. And there are patients who can't do much with it.” (FFM03)*  *“Hm. That wasn't a big deal. I mean, I write the prescription and then I get feedback that it works or not. But it usually works, I don't get any feedback during the process if I don't ask them myself […]”. (DD_GP07)* |
| **Attitudes towards DHA-MD** | - **Patient preference** - **Assessment of effectiveness** - **Importance of DHA-MD in primary care** - **Perceived need for DHA-MD in primary care** | *„So, it's always an individual and personal decision, but if you want to try [a DHA-MD], then you go for it […].” (DD_GP04)*  *“I would say it is significantly lower than for psychotherapy. But I do think that it can have a stabilizing effect, simply by providing something tangible and guiding you through it, just for aspects of reinforcement, for imparting knowledge about the disease.” (DD_GP07)*  *“Yes. Demand has increased now due to the pandemic. Hm. For mental health services. And I think that it can also be a support for society as a whole to avoid longer periods of sick leave in the economy. Or to shorten existing periods of sick leave. To ensure that people get back into the labor market faster. Hm. Yes. So, in terms of society as a whole, the DHAs could certainly also be of benefit, not only now to relieve the pressure on doctors' surgeries, but also directly in social terms.” (DD_GP05)*  *“To be honest, you wouldn't need them at all if there were enough therapy places. And enough medical capacity to be able to conduct the sessions yourself. Because I think that if we were all sufficiently trained and the therapists were available, it would be nice to be able to do a short therapy live and start it within the next few weeks.” (DD_GP02)* |

*Pseudonymization codes
