# Supplementary material for: General Practitioners’ Perspectives on Digital Health Applications for Mental Disorders and Their Prescribing Behavior: Mixed Methods Study
Source: JMIR Ment Health. 2026 Jan 6;13:e78659. doi: 10.2196/78659 (PMC12774394; doi:10.2196/78659)
Supplement: Multimedia Appendix 2 [file mental-v13-e78659-s002.docx]

## Multimedia Appendix 2. Results of interviews with medical assistants.

| **Main category** | **Subcategory** | **Example quote** |
| --- | --- | --- |
| **Experiences with DHA-MD** | - **Importance of DHA-MD** - **Difficulties with the integration of DHA** - **Information sources for DHA-MD** - **Prescription of DHA** - **Missing knowledge** - **Challenges in selecting suitable patients** | *“And it's also naturally a way to bridge the gap, especially in the psychological field. Until a spot with a psychologist is found, I do think it can be helpful.” (DD_MFA04*)*  *“That this is rather difficult for us to implement in rural areas. In our practice, we mainly have older patients who don't know what to do with digital health apps. And then it's also really very difficult with younger patients, because you have to filter out the appropriate clientele.” (DD_MFA03)*  *“Or brochures that the representatives give you. There are quite a few of those now. Yes. But, let me say, in the first place through the internet.” (DD_MFA04)*  *“In our practice, […] we now prescribe it for mental disorders, in patients who have nutritional problems or weight problems. We also prescribe it for patients who may be going through the menopause and have sleep problems. […]. (DD_MFA02)*  *“The app is still relatively unknown. So many patients and staff don't even know about it. Basically, it should be publicized more, there should be more advertising that something like this even exists.” (DD_MFA02)*  *“So actually, the most important thing is always to filter out the patients who are suitable for something like this. Because the inhibition threshold for talking about these problems, which one might have in everyday life or in a partnership or in an illness, is still a very high one. And that, I think, is the main problem, that patients open up to the doctor and perhaps first ask whether there is an option.” (DD_MFA03)* |
| **Tasks related to DHA** | - **No concrete tasks for MA** - **Document preparation** - **Prescription process** - **Reimbursement process** - **Interim report** | *“So, in my day-to-day work, it doesn't really affect me that much. It's really only done by the doctor. Because he has, so to speak, summoned the patients and is conducting the psychosomatic discussions with them.” (DD_MFA01)*  *“We prescribe them based on instructions, of course. The prescriptions are issued at our front desk, but that's probably more of an organizational matter, depending on how you handle it. For example, keeping flyers ready and preparing them if necessary. Patients sometimes open up to us about these issues. In such cases, we can already give the doctors a heads-up, so to speak.” (DD_MFA01)*  *“That is not really our job so far. And it has not been brought to our attention that support is being provided in this regard. No. No.” (DD_MFA01)*  *"At the moment, we remind the general practitioners to use the billing code that exists for this. Because it hasn’t really become routine yet. It’s all still relatively new." (DD_MFA01)*  *“We have not received any [interim reports] yet.” (DD_MFA01)* |
| **Questions of patients to DHA** | - **No concrete questions** - **General questions about DHA** - **Questions after medical consultation** | *“Well, they have never said anything to me about it. I don't even know if patients are aware of it.” (DD_MFA04)*  *“Hm. Patients ask if/what that is at all. They've read somewhere that it's a possibility. How it actually works. Whether you need a prescription or a recommendation. What actually happens on such an app, how it works. Or rather, what kind of DHA options are available. These are actually the questions we get from patients.” (DD_MFA02)*  *“Rather not […] because patients usually try it or are prescribed it for the first time in the practice. And our doctor, I think, explains it very well. I think the questions tend to come when people who are either already using the app come back to the doctor's office. But otherwise, they get this prescription with relatively little expectation and just go with it or see what happens.” (DD_MFA02)* |
| **Feedback of patients to DHA** | - **Negative feedback** - **No feedback to MA** - **Positive feedback** | *“Once so far, […]. But that was more of a negative [feedback]. That was someone who couldn't cope with it. The others are still in the middle of using the DHA. And there we are still waiting for feedback, so to speak. (DD_MFA01)*  *“Exactly. The feedback is then only given to the doctor in the same context. Because, you have to say, these are also psychological problems that are not discussed at the front desk.” (DD_MFA03)*  *“Yes. I have friends who have one, for example, and also a patient who uses it. And there was really positive feedback, […] especially with regard to a diet; it was for nutrition, and also for sleep problems during menopause. And there was really very, very good feedback. Where I myself was amazed, […] one would really have to involve it more in practice. Or rather, give patients the opportunity, the information, that something like this is possible at all.” (DD_MFA02)* |

*Pseudonymization codes.
